# Mechanistic study on the optimization of soil ecological structure and promotion of rapeseed (*Brassica napus* L.) growth by co-application of organic fertilizer and biochar

**DOI:** 10.3389/fmicb.2026.1874862

**Published:** 2026-06-25

**Authors:** Jiahao Jia, Junchi Ma, Shuo Liu, Yinbiao Zhou, Jinhui Jiang, Jindan Wang, Chaojun Shi, Neng Yang, Honglin Zhang, Lương Hùng Tiến, Hoàng Trung Tín, Ruilin Fu, Tao Liu, Yu Liu, Qian Li

**Affiliations:** 1 College of Agronomy and Biotechnology, Yunnan Agricultural University, Kunming, China; 2 Institute of Technology, Xinyang University, Xinyang, China; 3 College of Tobacco Science, Yunnan Agricultural University, Kunming, China; 4 Faculty of Agronomy, Thai Nguyen University of Agriculture and Forestry, Thai Nguyen, Vietnam; 5 GCP Department, Shenzhen Traditional Chinese Medicine Hospital, The Fourth Clinical Medical College of Guangzhou University of Chinese Medicine, Shenzhen, China; 6 The Key Laboratory of Agricultural Microbiome of Yunnan Province, Yunnan Agricultural University, Kunming, China

**Keywords:** biochar, community diversity, rapeseed, rhizosphere soil bacterial community, soil nutrients

## Abstract

Despite the continuous diversification of amelioration strategies for acidic red soils in recent years, a distinct research gap persists: the lack of data-driven quantitative evidence to elucidate the cascading response pathway linking soil physicochemical remodeling, rhizosphere microbial succession, and crop economic traits. To bridge this gap, we hypothesized that the co-application of organic fertilizers and biochar induces distinct, source-dependent responses in soil acidity neutralization and microbial community restructuring, thereby driving specific variations in crop performance. This study aimed to evaluate these responses and decipher key biological associations utilizing multivariate data models. Experimental data revealed that both individual and integrated regimes effectively mitigated acidic stress (elevating pH from 4.93 to 5.04–6.47) and substantially boosted organic matter content (18.24–73.82%). Furthermore, Principal Coordinate Analysis (PCoA) showed clear separation of rhizosphere bacterial communities among treatments, and ANOSIM further confirmed significant differences in community structure (*R* = 0.881, *p* = 0.001). Crucially, crop agronomic outcomes exhibited strong source-dependent patterns rather than a uniform superiority of combined applications. Subsequent statistical association analyses pinpointed that specific integrated treatments enriched the phylum Firmicutes, whose relative abundance exhibited a highly significant positive coupling with seed oil accumulation (*r* = 0.838, *p* < 0.05). Ultimately, this study provides a novel strategic framework and robust data support for the precision management of fertilization in rapeseed (*Brassica napus* L.) production within acidic red soil ecosystems.

## 1 Introduction

Fertilization, as one of the most important management practices in agricultural production, not only directly influences nutrient supply for crops but also regulates nutrient cycling processes by altering soil microbial community structure (Adesemoye and Kloepper, 2009; Hu et al., 2024). However, the long-term sole application of chemical fertilizers has led to nutrient imbalances and exacerbated soil acidification, thereby inhibiting normal growth and development (Noor et al., 2020). Moreover, excessive fertilization is closely associated with a range of serious environmental problems, particularly the leaching of nutrients such as nitrogen and phosphorus, which can enter aquatic ecosystems and induce eutrophication, thereby posing a threat to ecosystem stability (Yadav et al., 2013). In this context, the identification of fertilization strategies that simultaneously enhance crop productivity while minimizing environmental impacts has become an important focus of contemporary agricultural research (Le et al., 2023).

Rapeseed (*Brassica napus* L.), as the most important oil-seed crop in China, has its yield and quality directly linked to national edible oil security (Fu et al., 2016). The Yangtze River Basin, being the largest rapeseed-producing area in the country (Geng et al., 2021), has soil fertility conditions that play a decisive role in rapeseed production. Yunnan Province, located in the ecologically fragile upper reaches of the Yangtze River, faces widespread soil problems such as acidification (pH < 5.5), organic matter deficiency, and insufficient micronutrient availability (Liang et al., 2022), which severely constrain high-yield and high-quality rapeseed production. The traditional chemical fertilization model in this region not only offers limited yield optimization but may also exacerbate soil degradation. Therefore, exploring green fertilization techniques suitable for acidic red soil regions is of great strategic significance for ensuring the sustainable development of the regional rapeseed industry (Feng et al., 2020; Luo et al., 2018).

In recent years, biochar has shown great potential in soil optimization due to its unique porous structure and surface properties. Biochar can not only slow down the release of nutrients from organic fertilizers and enhance their utilization efficiency (Alling et al., 2014), but also improve the soil environment by increasing cation exchange capacity (CEC) and providing additional habitats for microorganisms (Kang et al., 2022; Mukherjee et al., 2022).

Biochar is produced through the pyrolysis of diverse organic materials, with agricultural residues representing one of the major feedstock sources, and exhibits multifunctional potential in improving soil health, enhancing carbon sequestration, and remediating contaminated soils and water bodies. Its porous structure and capacity to enhance soil microaggregate stability provide suitable habitats for microorganisms, while also serving as a source of carbon and nutrients. Moreover, by regulating soil physicochemical properties, biochar creates favorable conditions for the maintenance and enhancement of microbial diversity (Bolan et al., 2024; Hoque et al., 2025). Recent studies have further shown that biochar-based integrated amendments can regulate rhizosphere microbiomes, improve nutrient availability, and enhance plant stress resilience. For example, bacterial-charged biochar was reported to increase nitrogen and phosphorus availability and enrich beneficial microbial taxa, while nano-engineered biochar composites improved soil microbial interactions and plant stress-response pathways under contaminated soil conditions (Afzal et al., 2025; Yasin et al., 2025). However, whether similar soil–microbe–plant linkages occur in acidic red soils under different organic fertilizer sources remains insufficiently understood.

Organic fertilizers, rich in organic matter and various nutrients, are considered effective alternatives to chemical fertilizers. Studies have shown that organic fertilizers can promote crop growth by improving soil aggregate structure and enhancing water and nutrient retention capacity (Plaza et al., 2016; Wato et al., 2024). However, the sole application of organic fertilizers presents certain limitations, such as an asynchrony between nutrient release rates and crop nutrient demand, and, over the long term, may lead to issues such as heavy metal accumulation and microbial community dysbiosis (Adhikari et al., 2020; Ma et al., 2022; Niedziński et al., 2021; Wan et al., 2016).

In the acidic red soil regions of Yunnan Province, China, research on the co-application of organic fertilizers and biochar in rapeseed production still faces several key knowledge gaps that require urgent elucidation. Specifically, it is necessary to determine whether different sources of organic fertilizers (e.g., sugarcane bagasse, sheep manure, and nutshells) combined with biochar exert varying, source-dependent effects on the amelioration of acidic red soils. Furthermore, the mechanisms by which this combined application model influences rapeseed yield and quality—particularly through the regulation of rhizosphere microbial communities and key functional taxa such as Firmicutes—remain poorly understood. Finally, there is a critical need to clarify the interactive relationships and cascading responses between changes in soil physicochemical properties and the succession of microbial communities.

In this study, the main rapeseed cultivar in Yunnan, ‘*Huayou 8*’, was used as the experimental material to systematically investigate the effects of three types of organic fertilizers (sugarcane bagasse, sheep manure, and Australian macadamia nutshell) applied alone or in combination with biochar on rapeseed growth, soil properties, and microbial communities. By integrating agronomic trait analysis, soil chemical characterization, and high-throughput sequencing technologies, the study focused on: (i) elucidating the mechanisms by which the co-application of organic fertilizer and biochar ameliorates acidic red soils; (ii) clarifying the relationships between changes in rhizosphere microbial community structure and the formation of rapeseed yield and quality. The findings will provide a theoretical basis and technical support for green and high-yield rapeseed cultivation in ecologically fragile areas of Yunnan Province, and hold significant practical value for promoting sustainable agricultural development.

## 2 Materials and methods

### 2.1 Materials

Test variety: Test organic fertilizers included sugarcane bagasse organic fertilizer purchased from Shijiazhuang Wofowo Fertilizer Co., Ltd. (Hebei, China), sheep manure organic fertilizer obtained from Shenyang Beinong Biotechnology Co., Ltd. (Liaoning, China), and Australian macadamia nutshell organic fertilizer procured from Yunnan Disi Enterprise Group Macadamia Co., Ltd. (Yunnan, China). All organic fertilizers were produced using conventional aerobic composting processes prior to application.

Biochar: The biochar was produced from maize straw and supplied by Henan Xingnuo Environmental Protection Materials Co., Ltd. (Henan, China). The biochar used in this study was produced by slow pyrolysis under oxygen-limited conditions, a method widely adopted for agricultural biochar production. According to information provided by the manufacturer, the biomass feedstock was pyrolyzed at approximately 500 °C, followed by natural cooling under an inert atmosphere (Yuan et al., 2011).

Representative subsamples of the organic fertilizers and biochar were air-dried under shaded conditions, ground, and passed through 20-mesh (0.84 mm) and 100-mesh (0.15 mm) sieves. The pH, organic matter content, and total N, P, and K contents were determined. Materials applied to the soil were not oven-dried at high temperature in order to avoid alterations to their biological and chemical properties. The main component contents are presented in Table 1.

**Table 1:** Physicochemical properties of the biochar and organic fertilizers used in the experiment.

Materials	pH	SOM	TN	TP	TK	AN	AP	AK
		(g/kg)	(g/kg)	(g/kg)	(g/kg)	(mg/kg)	(mg/kg)	(mg/kg)
Biochar	9.47 ± 0.02b	297.11 ± 1.43c	2.58 ± 0.02d	0.50 ± 0.01a	11.24 ± 0.13d	63.85 ± 1.25d	14.19 ± 0.12d	0.76 ± 0.01d
Sugarcane bagasse organic fertilizer	10.05 ± 0.03a	305.60 ± 0.65b	17.53 ± 0.78a	2.64 ± 0.06b	21.34 ± 0.19a	727.81 ± 1.64b	397.50 ± 1.78a	2.04 ± 0.30a
Sheep manure organic fertilizer	7.49 ± 0.03c	307.66 ± 3.97b	16.1 ± 0.44b	4.62 ± 0.01c	14.15 ± 0.22c	414.98 ± 1.61c	393.14 ± 3.10b	1.26 ± 0.06c
Australian macadamia nutshell organic fertilizer	7.06 ± 0.02d	347.74 ± 1.01a	14.68 ± 0.58c	5.64 ± 0.07d	15.73 ± 0.21b	903.79 ± 0.40a	330.16 ± 2.00c	1.43 ± 0.02b

Data are expressed as mean ± standard deviation (SD) (*n* = 3). Different lowercase letters within the same column indicate significant differences among materials at *p* < 0.05 according to Duncan’s multiple range test. SOM, soil organic matter; TN, total nitrogen; TP, total phosphorus; TK, total potassium; AN, available nitrogen; AP, available phosphorus; AK, available potassium.

### 2.2 Description of the experimental site

Soil conditions: The field experiment was conducted at the experimental farm of Yunnan Agricultural University (25°13′N, 102°75′E) at an altitude of approximately 1925.5 MSL, with maize as the preceding crop. Within the experimental field, 15 sampling points were evenly distributed in an “S” pattern, and topsoil samples (0–20 cm tillage layer) were collected and stored in sealed bags. In the laboratory, the samples were air-dried under shaded conditions, ground, and passed through 20-mesh (0.84 mm) and 100-mesh (0.15 mm) sieves for physicochemical analysis. Before sowing, the soil had a pH of 4.93 and an organic matter content of 28.35 g kg^−1^. The contents of total nitrogen, total phosphorus, and total potassium were 1.48, 1.43, and 7.38 g kg^−1^, respectively, while the concentrations of available nitrogen, available phosphorus, and available potassium were 135.3, 9.75, and 160 mg kg^−1^, respectively.

### 2.3 Experimental design and field management

The field experiment comprised eight treatments: CK, no fertilizer application (control); C (biochar alone); BO (sugarcane residue organic fertilizer alone); SO (sheep manure organic fertilizer alone); MO (Australian green-husked macadamia shell organic fertilizer alone); BOC (sugarcane residue organic fertilizer combined with biochar); SOC (sheep manure organic fertilizer combined with biochar); MOC (Australian green-husked macadamia shell organic fertilizer combined with biochar). Each treatment was replicated three times in a completely randomized block design. Each plot measured 4 m^2^, with a 1 m buffer between plots. The application rates of biochar and organic fertilizers when applied alone followed the recommended rate of 2 kg/m^2^. For combined applications, organic fertilizer and biochar were mixed at a 1:1 ratio, with a total amendment rate of 2 kg m^−2^. Rapeseed was planted at a density of approximately 10,000 plants per hectare. Fertilizers were applied using the hole application method according to local agronomic practices for rapeseed cultivation (Chen et al., 2024).

### 2.4 Measurement of parameters and methodology

#### 2.4.1 Determination of major agronomic traits, yield, and quality of rapeseed at harvest

At the harvest stage, nine representative rapeseed plants were selected from each treatment (three plants per replicate, with three replicates) to measure key agronomic traits, including plant height, root collar diameter, root length, and aboveground dry biomass.

TSW (thousand seed weight): Randomly collected seeds were oven-dried at 40 °C to constant weight and weighed in groups of 1,000 seeds using a high-precision electronic balance (Geng et al., 2025).

Yield: Three representative plants per plot were harvested for siliques, which were air-dried at 25 °C until dehiscent. Seeds were manually threshed, cleaned by sieving, weighed, and the average yield per plant was calculated.

Oil content determined: Oven-dried and ground rapeseed samples were extracted using a Soxhlet apparatus with petroleum ether (boiling range 60–90 °C) for 6–8 h. Oil content was calculated gravimetrically following the National Standard of the People’s Republic of China (GB 2906-82) (Aytaç, 2021).

Protein content determination: An accurately weighed portion of dried and ground rapeseed sample (approximately 0.3–0.5 g) was transferred into a digestion tube and digested with concentrated sulfuric acid in the presence of a catalyst until the solution became clear. After cooling, the digest was subjected to distillation and titration using a Kjeldahl nitrogen analyzer to determine total nitrogen content. Crude protein content was calculated from the measured nitrogen concentration using the standard conversion factor (Shaw and Beadle, 2021).

#### 2.4.2 Soil chemical properties

After rapeseed harvest, soil samples were collected from the 0–20 cm plow layer of each plot and placed in sealed bags. The samples were transported to the laboratory, air-dried in the shade, ground, and passed through 20-mesh (0.84 mm) and 100-mesh (0.15 mm) sieves, and then stored at 4 °C for subsequent soil chemical analyses. Soil chemical properties were determined by Sanshuo Biotechnology Co., Ltd. (Yunnan, China) (Nyamasoka-Magonziwa et al., 2020).

#### 2.4.3 Rhizosphere soil sampling and microbiome sequencing

Plants were carefully uprooted, and tightly adhering rhizosphere soil was collected using sterile brushes. Approximately 5–10 g of soil was pooled from multiple points per plot into sterile bags and transported on ice. In a sterile laminar flow hood, debris was removed, and soil was passed through a 2 mm sieve before being aliquoted into 20 mL sterile tubes (10 g per tube) (Liu et al., 2022). Samples were flash-frozen in liquid nitrogen and stored at −80 °C before shipment to Wuhan Frasergen Gene Information Co., Ltd. (Hubei, China). For 16S rRNA sequencing.

Total soil DNA was extracted, and full-length primers with barcodes were synthesized for PCR amplification. PCR products were purified, quantified, and normalized to construct SMRTbell sequencing libraries (Callahan et al., 2019). The libraries underwent quality control, and those passing QC were sequenced on the PacBio Sequel II platform (Kavamura et al., 2018). Circular consensus sequencing (CCS) reads were extracted from the raw data. Barcode identification of CCS reads was performed using lima v1.7.0 (Gaudin et al., 2023), and primer sequences were recognized and removed, followed by length filtering using cutadapt 1.9.1 (Feng et al., 2024), generating primer-free clean CCS reads (Clean-CCS). Chimeric sequences were identified and removed using UCHIME v4.2 (Chen et al., 2022), resulting in effective CCS sequences (Effective-CCS). After quality filtering, the number of effective reads per sample ranged from 47,544 to 61,991, with a median of 54,995 reads. Effective CCS sequences were clustered into operational taxonomic units (OTUs) based on sequence similarity, and taxonomic classification was assigned based on representative feature sequences (Singer et al., 2016). The sequencing data have been deposited in the NCBI Sequence Read Archive (SRA) under BioProject accession number PRJNA1345503.

### 2.5 Data processing and analysis

Data on rapeseed traits, yield, quality, and soil chemical properties were compiled and processed using Excel 2021. Statistical analyses were performed using IBM SPSS Statistics 27 (SPSS, Chicago, USA). One-way analysis of variance (ANOVA) was used to assess significant differences among treatments (*n* = 3, *p* < 0.05). Graphs were generated using Origin 2022. Alpha and beta diversity indices of rhizosphere soil bacterial communities were analyzed using QIIME2 2020.6 (Bolyen et al., 2019).

## 3 Results

### 3.1 Growth, yield, and quality responses of rapeseed are highly dependent on organic fertilizer sources

At the harvest stage of rapeseed, all agronomic traits under fertilization treatments were significantly higher than those of the CK (Table 2). However, the effects of biochar co-application varied substantially depending on the organic fertilizer source. Compared with the single BO treatment, the combined application with BOC increased root length and aboveground dry weight by 6.65 and 8.91%, respectively. Conversely, while the SOC treatment enhanced aboveground dry weight (13.12%) and the number of siliques per plant (10.62%) relative to SO, the MOC treatment only resulted in a marginal increase in plant height (2.21%) compared to MO.

**Table 2:** Major agronomic traits of rapeseed at harvest under different treatments (*p* < 0.05; *n* = 9).

Treatments	Plant height (cm)	Root collar diameter (mm)	Root length (cm)	Aboveground dry biomass (g/plant)	Number of effective branches (branches/plant)	Main inflorescence silique length (cm)	Number of siliques per plant	Number of seeds per silique
CK	74.31 ± 8.44e	15.60 ± 0.91c	14.82 ± 1.50 e	42.54 ± 3.29f	4.44 ± 0.73d	6.59 ± 0.59c	176.89 ± 9.27f	25.11 ± 2.09d
C	117.71 ± 11.79bcd	16.37 ± 1.50 c	20.54 ± 1.32b	52.14 ± 2.70e	7.00 ± 1.00bc	8.18 ± 0.48ab	249.11 ± 12.67e	31.89 ± 3.69a
BO	118.67 ± 4.97bcd	18.72 ± 1.02b	18.39 ± 1.40d	61.07 ± 3.00c	7.67 ± 1.41ab	8.23 ± 0.55a	290.33 ± 11.72bc	29.00 ± 1.66bc
SO	115.34 ± 4.77 cd	18.04 ± 1.15b	18.42 ± 0.79d	57.244 ± 2.12d	6.67 ± 0.71c	8.11 ± 0.55ab	269.67 ± 11.28d	27.11 ± 2.15 cd
MO	124.98 ± 8.61ab	21.62 ± 1.04a	21.90 ± 1.94a	84.39 ± 4.01a	8.56 ± 1.59a	8.02 ± 0.39ab	340.56 ± 18.62a	32.56 ± 3.64a
BOC	122.00 ± 10.17abc	18.49 ± 1.03b	19.70 ± 2.12c	67.04 ± 2.12b	7.44 ± 1.01bc	7.94 ± 0.48ab	299.11 ± 9.18b	31.56 ± 2.79ab
SOC	113.53 ± 9.49 d	18.60 ± 1.42b	19.1 ± 1.30 cd	65.89 ± 2.66b	6.89 ± 1.27bc	7.72 ± 0.82b	270.11 ± 8.37d	30.33 ± 2.96ab
MOC	127.8 ± 10.18 a	17.71 ± 1.73b	21.83 ± 1.03a	66.21 ± 2.97b	6.56 ± 1.01c	8.32 ± 0.54a	285.56 ± 11.64c	31.78 ± 3.56a

Data are expressed as mean ± standard deviation (SD) (*n* = 9). Different lowercase letters within the same column indicate significant differences among treatments at *p* < 0.05 according to Duncan’s multiple range test.

Measurements of yield and quality-related parameters further highlighted these source-dependent responses (Table 3). Although TSW and total yield in all fertilization treatments were significantly higher than those of the CK, the co-application of biochar did not consistently outperform individual organic amendments in terms of crop yield. Specifically, while the BOC treatment significantly increased TSW and single-plant yield by 11.43 and 20.40% compared with BO, single-plant yield under the MO treatment was actually higher than that under MOC, and the SO treatment yielded slightly higher than SOC. Regarding seed quality, the SOC treatment enhanced seed oil content by 13.87% relative to SO, and the MOC treatment increased oil and protein contents by 12.91 and 8.93% relative to MO. In contrast, BOC showed no significant changes in these quality traits compared to BO.

**Table 3:** Yield and quality of rapeseed at different harvest stages (*p* < 0.05; *n* = 3).

treatments	TSW (g)	Yield per plant (g)	Oil content (%)	Protein content (mg/g)
CK	2.12 ± 0.07d	8.60 ± 0.30f	33.11 ± 2.95c	149.07 ± 20.22c
C	2.98 ± 0.21c	13.92 ± 0.76e	35.06 ± 4.21bc	160.63 ± 25.66abc
BO	3.10 ± 0.14bc	15.96 ± 0.74d	42.57 ± 1.98a	185.50 ± 5.00a
SO	3.34 ± 0.16ab	13.59 ± 0.76e	36.44 ± 2.57bc	152.70 ± 8.81bc
MO	2.99 ± 0.22c	21.47 ± 0.72a	33.59 ± 1.05c	147.17 ± 9.92c
BOC	3.50 ± 0.08a	20.05 ± 0.72b	41.82 ± 1.72a	179.77 ± 3.92ab
SOC	3.52 ± 0.23a	13.41 ± 0.98e	42.31 ± 2.25a	139.60 ± 10.91c
MOC	3.08 ± 0.16bc	17.95 ± 0.47c	38.57 ± 2.00ab	161.60 ± 23.81abc

TSW, thousand seed weight. Data are expressed as mean ± standard deviation (SD) (*n* = 3). Different lowercase letters within the same column indicate significant differences among treatments at *p* < 0.05 according to Duncan’s multiple range test.

### 3.2 Organic amendments and their combinations with biochar differentially remodel acidic soil pH and fertility

**Figure 1:**
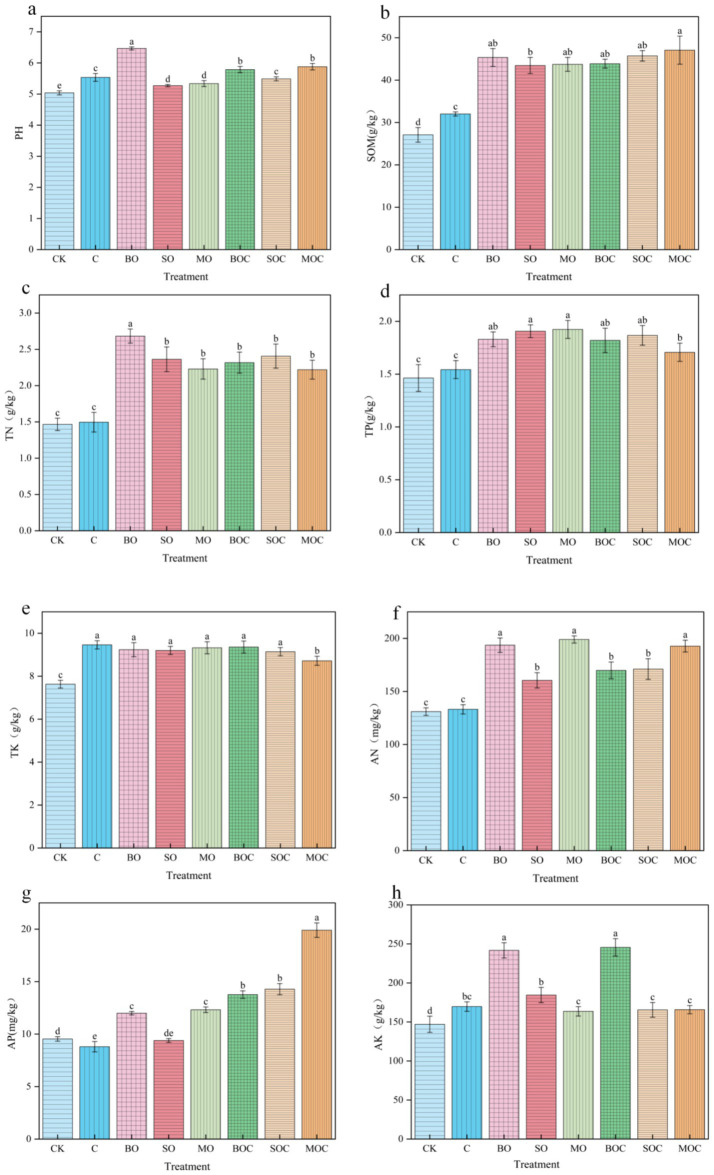
**(a)** Effects of different treatments on soil pH; **(b)** effects of different treatments on soil organic matter (SOM) content; **(c–e)** effects of different treatments on soil total nitrogen, total phosphorus, and total potassium; **(f–h)** effects of different treatments on soil available nitrogen, available phosphorus, and available potassium (*p* < 0.05; *n* = 3). SOM, soil organic matter; TN, total nitrogen; TP, total phosphorus; TK, total potassium; AN, available nitrogen; AP, available phosphorus; AK, available potassium.

The results of soil pH and SOM content are presented in Figure 1. Soil pH values under all fertilization treatments were significantly higher than those of the CK (Figure 1a). Among the single applications, the BO treatment showed the highest value, increasing by 28.30% compared with CK. The co-application of biochar further modified soil acidity, with the SOC and MOC treatments increasing soil pH by 4.17 and 10.18%, respectively, relative to their corresponding single treatments. Regarding SOM, all treatments markedly enhanced soil organic matter content compared with CK, with increments ranging from 18.24 to 73.82% depending on the amendment type (Figure 1b).

For total soil nutrients, except for the C treatment, TN content in all fertilization treatments was significantly higher than that in CK (Figure 1c), with the BO treatment showing the highest increase of 82.95%. TP content was also elevated across all fertilization treatments relative to CK (Figure 1d). Similarly, TK content in all fertilization treatments was significantly greater than that in CK, with increases ranging from 14.29 to 24.03% (Figure 1e).

With respect to available nutrients, the responses also varied by fertilizer source. The MO treatment significantly increased available nitrogen (AN) content by 51.85% compared with CK, whereas the SOC treatment enhanced AN by 6.62% relative to SO (Figure 1f). Available phosphorus (AP) content in the BO, MO, BOC, SOC, and MOC treatments exceeded that in CK, and the combined applications of organic fertilizer with biochar showed significantly higher AP levels than their respective single organic fertilizer treatments (Figure 1g). All fertilization treatments significantly improved available potassium (AK) content relative to CK, with increases ranging from 11.32 to 67.02% (Figure 1h).

### 3.3 Alpha diversity analysis

**Figure 2:**
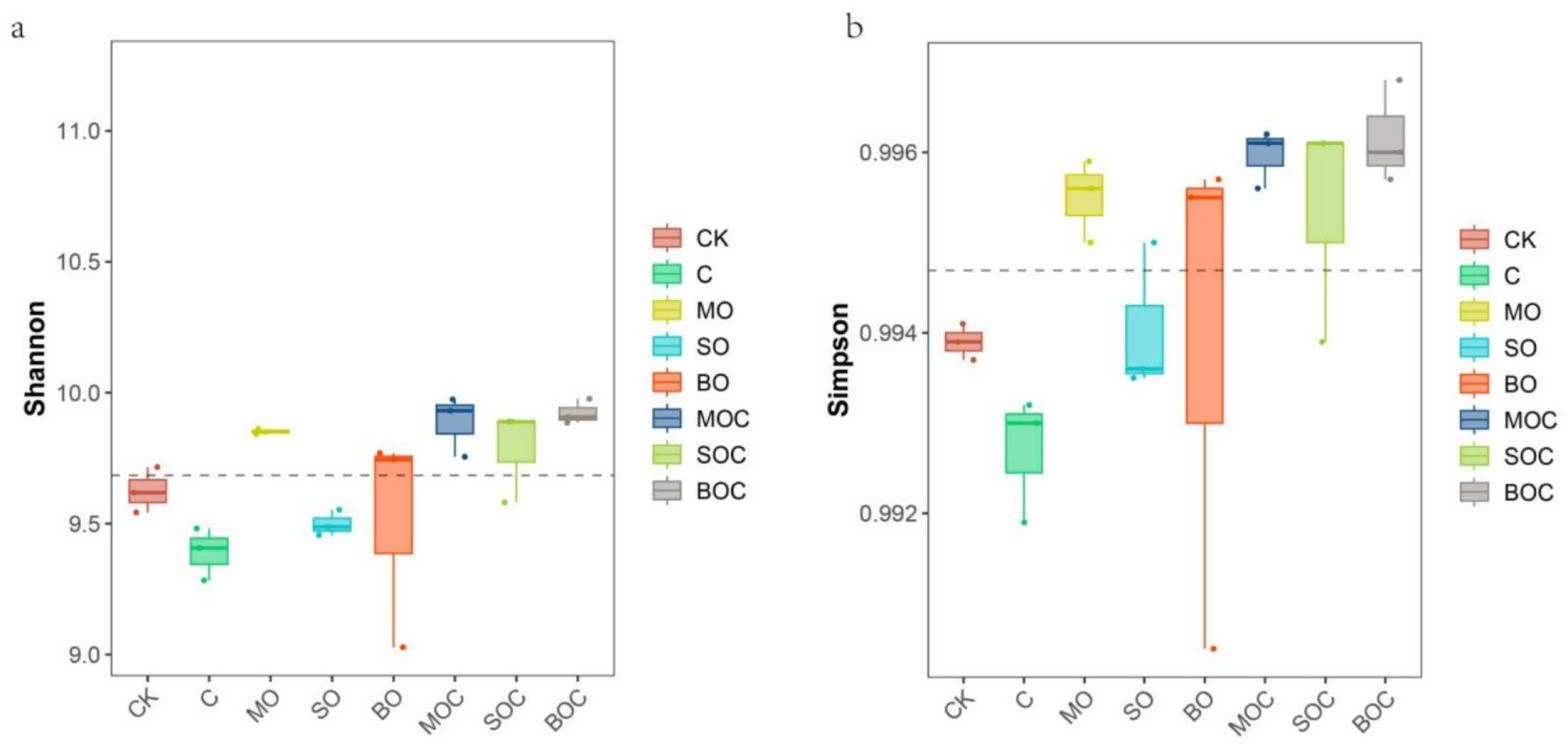
Boxplot of differences in alpha diversity indices among treatments. **(a)** Shannon index. **(b)** Simpson index. Alpha diversity was assessed using the Shannon–Wiener index and Simpson index to evaluate differences in bacterial community richness and diversity among treatments. The *x*-axis represents treatment groups, and the *y*-axis indicates the corresponding values of alpha diversity indices. The numbers shown above the connecting lines between boxes represent *p*-values from the *t*-test (*p*-values > 0.05 are not displayed by default).

Various combinations of applications demonstrated unique impacts on the growth, yield, and quality of rapeseed. To further elucidate these effects, an alpha diversity analysis of soil bacterial communities under different treatments was performed. The results revealed that, compared with the sole application of organic fertilizer or biochar, the combined application of both significantly enhanced the species richness and diversity of soil bacterial communities, as evidenced by notable increases in the Shannon and Simpson indices (Figure 2). Specifically, bacterial diversity in the BOC, SOC, and MOC treatments was higher than that in the CK, and the diversity indices of the combined treatments consistently exceeded those of their corresponding single-fertilizer treatments. These findings suggest that soil bacterial communities respond more sensitively to the integrated application of organic fertilizer and biochar, with community diversity and richness exhibiting more pronounced optimizations under the combined treatments.

### 3.4 Beta diversity analysis

**Figure 3:**
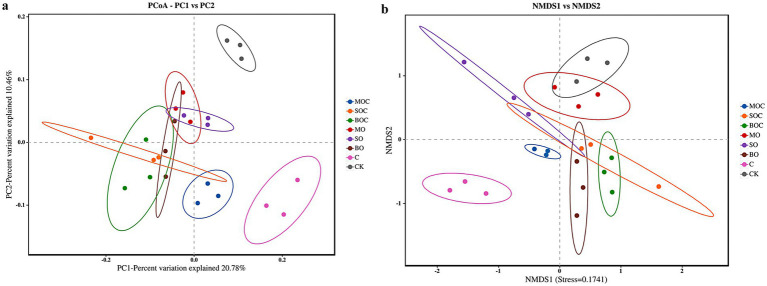
PCoA analysis and NMDS analysis. Each point in **(a)** represents a sample; the elliptical circle represents a 95% confidence ellipse; the percentage represents the contribution of principal components to sample differences. **(b)** Shows NMDS analysis. It is generally believed that when stress is less than 0.2, it indicates that NMDS analysis has a certain level of reliability. The closer the distance between each process on the coordinate graph, the higher the similarity.

The combined application of organic fertilizer and biochar altered the bacterial community structure in the rapeseed rhizosphere soil, with combined treatments showing distinct community shifts compared with single-amendment treatments. The results of both PCoA and Non-metric Multidimensional Scaling (NMDS) analyses were consistent (Figure 3), showing clear separation between all treatment groups and the control. Moreover, the combined application treatments were distinctly separated from the single-application treatments in the ordination space, further confirming that the combined application exerted a more pronounced impact on the rhizosphere microbial community structure.

**Figure 4:**
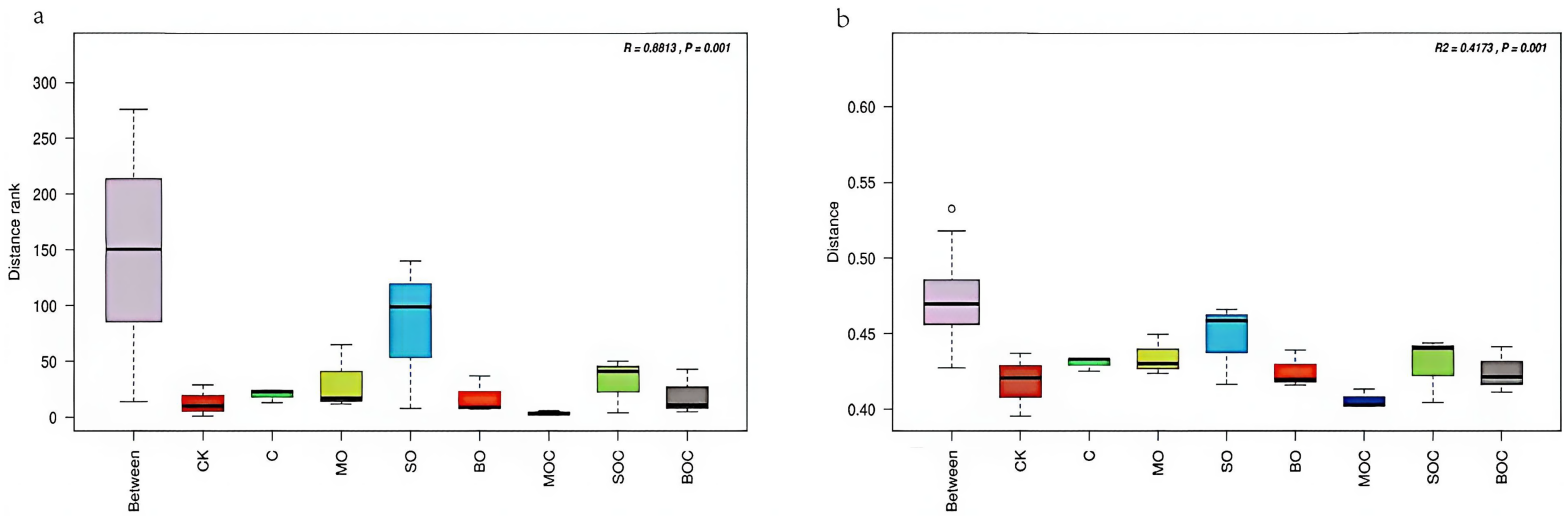
Comparison of differences between treatments based on Anosim/PERMANOVA analysis. R in **(a)** (*R* > 0.75: significant difference); *R* > 0.5: Moderate difference, *R* > 0.25: A small difference (*R* = 0 or around 0) indicates that there is no difference between the groups, and a *p* value less than 0.05 indicates high reliability of the test. The larger the *R*^2^ in **(b)**, the higher the explanatory power of the grouping for the differences, indicating greater grouping differences. When the *p* value is less than 0.05, it indicates high reliability of the test.

The results of ANOSIM and PERMANOVA analyses further demonstrated that the rhizosphere soil bacterial community structures differed significantly among treatments (*R* = 0.881, *p* = 0.001; *R*^2^ = 0.417, *p* = 0.001) (Figure 4a). The PERMANOVA results further validated this trend (Figure 4b). The consistency of the two tests indicates that different fertilization treatments exerted significant effects on the bacterial community structure.

### 3.5 Enrichment of beneficial microorganisms by combined application of organic fertilizer and biochar

**Figure 5:**
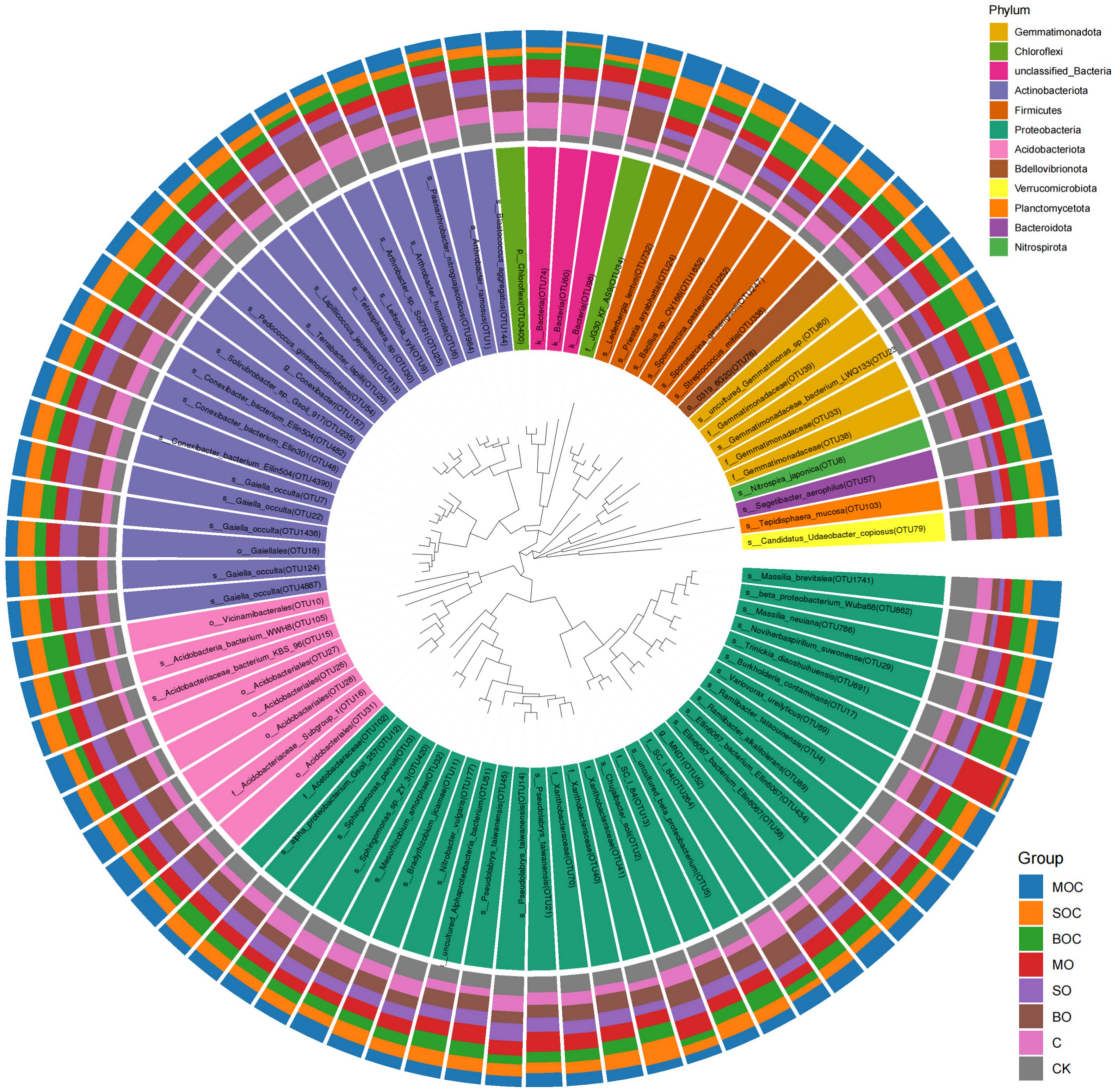
Species Evolutionary Tree and Distribution of Bacterial Communities at the phylum Level. The legend in the upper right corner shows the horizontal species names of the phylum, while the inner circle represents the evolutionary tree of species. Species with the same color in the inner circle represent the same phylum, and the outer circle represents the relative abundance proportion of the species in different treatments.

To elucidate the influence of rhizosphere microbial shifts following combined applications on rapeseed traits, the top 80 most abundant OTU at the phylum level were selected for multiple sequence alignment and phylogenetic tree construction (Figure 5.). The combined application of organic fertilizer and biochar resulted in a more diverse and balanced distribution of microbial communities in the outer circular branches, distinctly separated from those observed under single-application treatments and the control (CK). This pattern suggests that the combined application markedly restructured the phylogenetic composition of dominant taxa, thereby enhancing both community diversity and functional potential. Among all treatments, SOC displayed the most significant enrichment of dominant OTUs, particularly those associated with functional microbial groups. This restructuring of the microbial community not only strengthened soil nutrient cycling and organic matter decomposition capacity but also established a robust microbiological foundation for improving soil health, increasing crop yield, and enhancing stress resilience.

**Figure 6:**
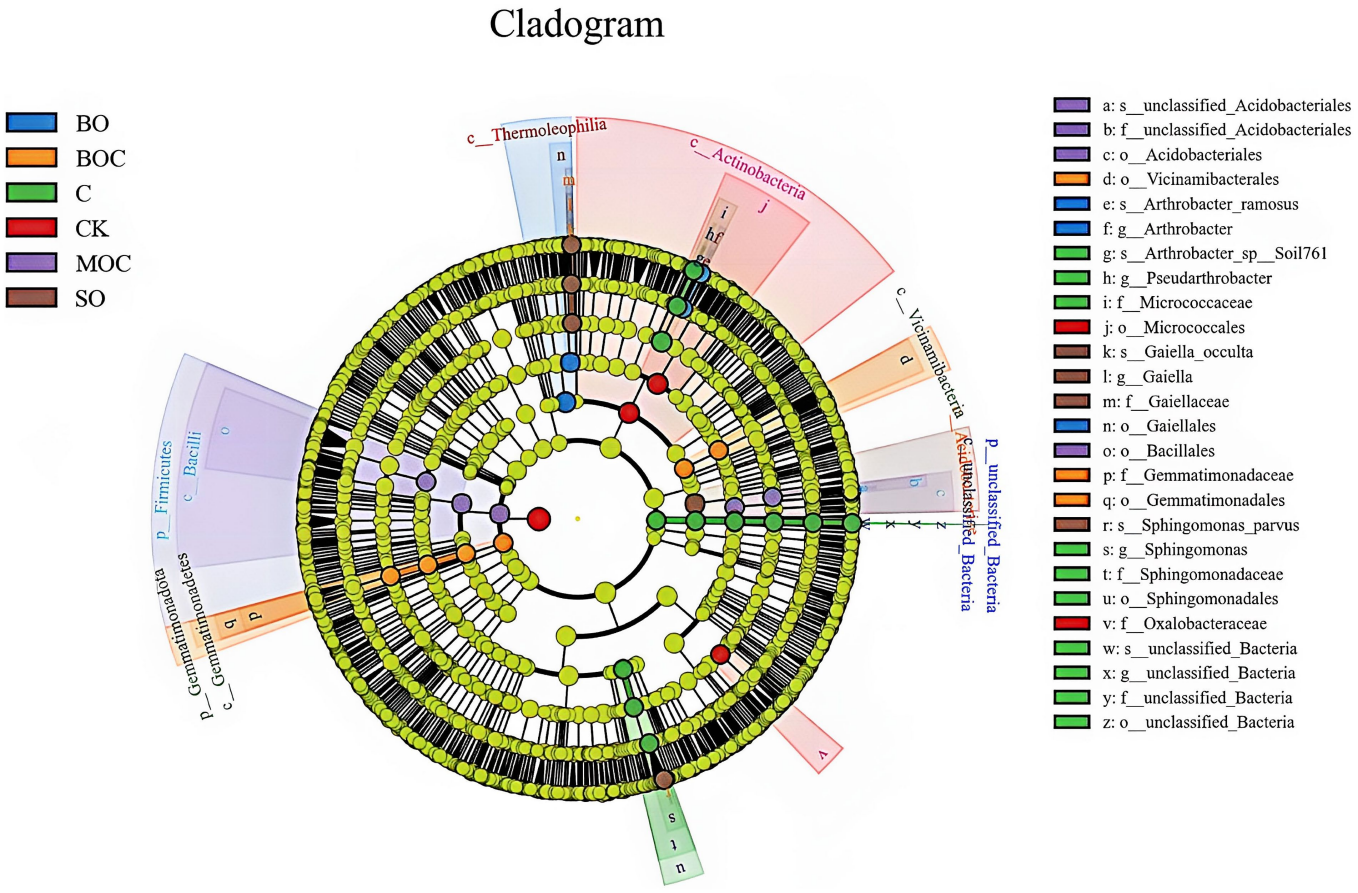
LEfSe analysis evolutionary branching diagram. The evolutionary cladogram represents taxonomic levels from phylum to species from the inside out. Each small circle at different taxonomic levels denotes a taxon at that level. Taxa without significant differences are uniformly colored in yellow, while other differential taxa are colored according to the group in which their abundance is highest.

To further verify the differences in rhizosphere soil aggregation under various combined treatments, Linear Discriminant Analysis Effect Size (LEfSe) analysis was performed to identify differential bacterial taxa among treatments (Figure 6). The results demonstrated that the treatments markedly altered the microbial community structure, with several taxa within the phyla Actinobacteria and Proteobacteria showing significant enrichment in specific treatment groups. These findings indicate that the combined application strategies exerted a substantial regulatory influence on the composition of soil microbial communities, thereby potentially affecting their ecological functions and contributions to soil health.

**Figure 7:**
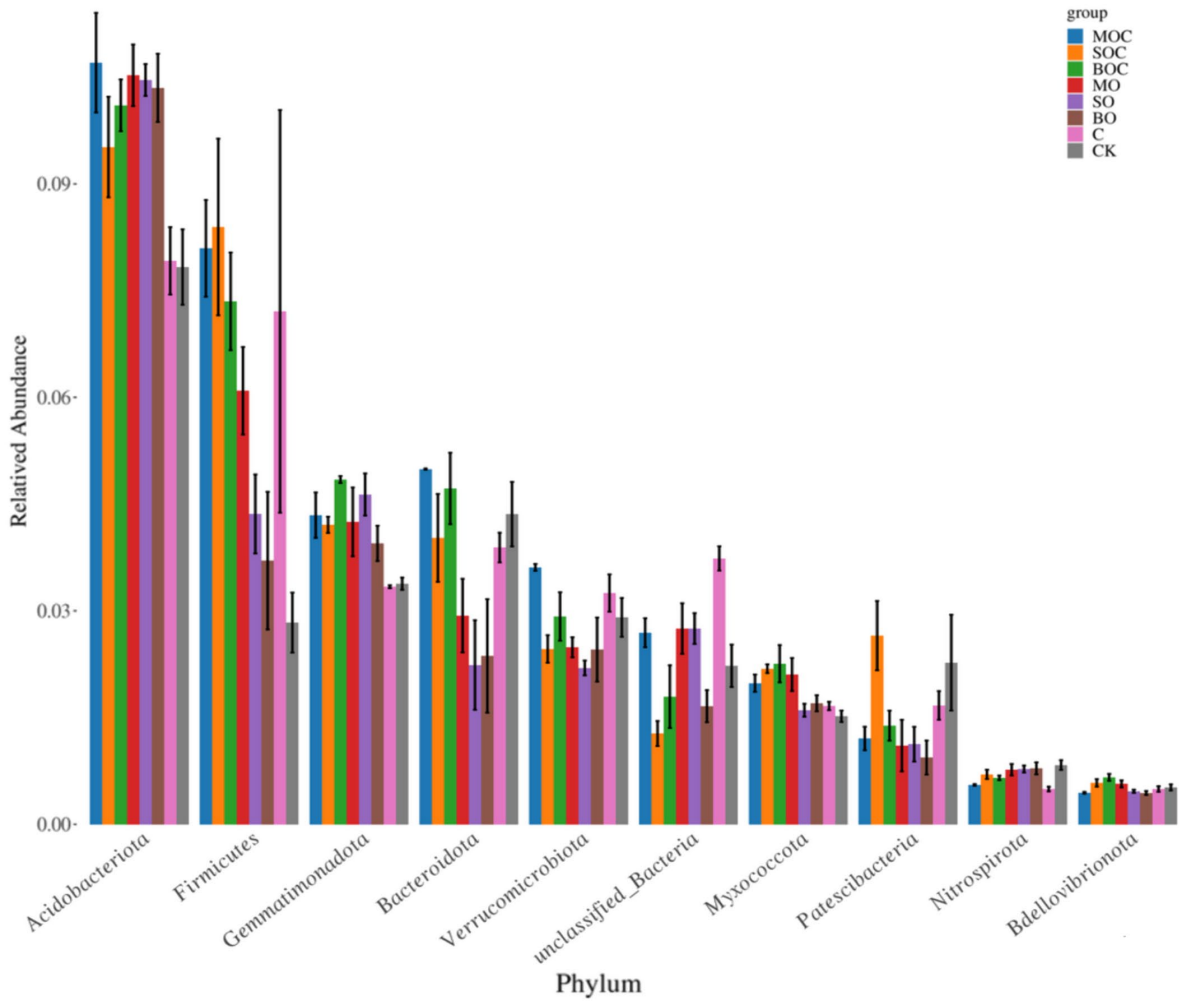
ANOVA between horizontal groups (*p* < 0.05, *n* = 3). Relative abundance of the top 10 dominant bacterial phyla across different treatments. The horizontal axis represents bacterial phyla, and the vertical axis represents their relative abundance. Bars indicate mean values, and error bars represent standard deviations.

To further analyze the effects of combined applications on soil bacterial communities and the differences among treatments, ANOVA was conducted to examine intergroup variations in species abundance (Figure 7). The 10 bacterial phyla showing the most significant differences among groups were identified. Compared with the single organic fertilizer treatments, Acidobacteriota, Bacteroidota, and Verrucomicrobiota were enriched in the MOC treatment relative to MO; Firmicutes and Patescibacteria were significantly enriched in the SOC treatment compared with SO; and Gemmatimonadota, Myxococcota, and Bdellovibrionota were enriched in the BOC treatment relative to BO. Unclassified bacteria showed the highest abundance in the C treatment, while Nitrospirota was enriched in the CK group. Notably, Actinobacteriota and Firmicutes exhibited the strongest responses to the combined application treatments.

**Figure 8:**
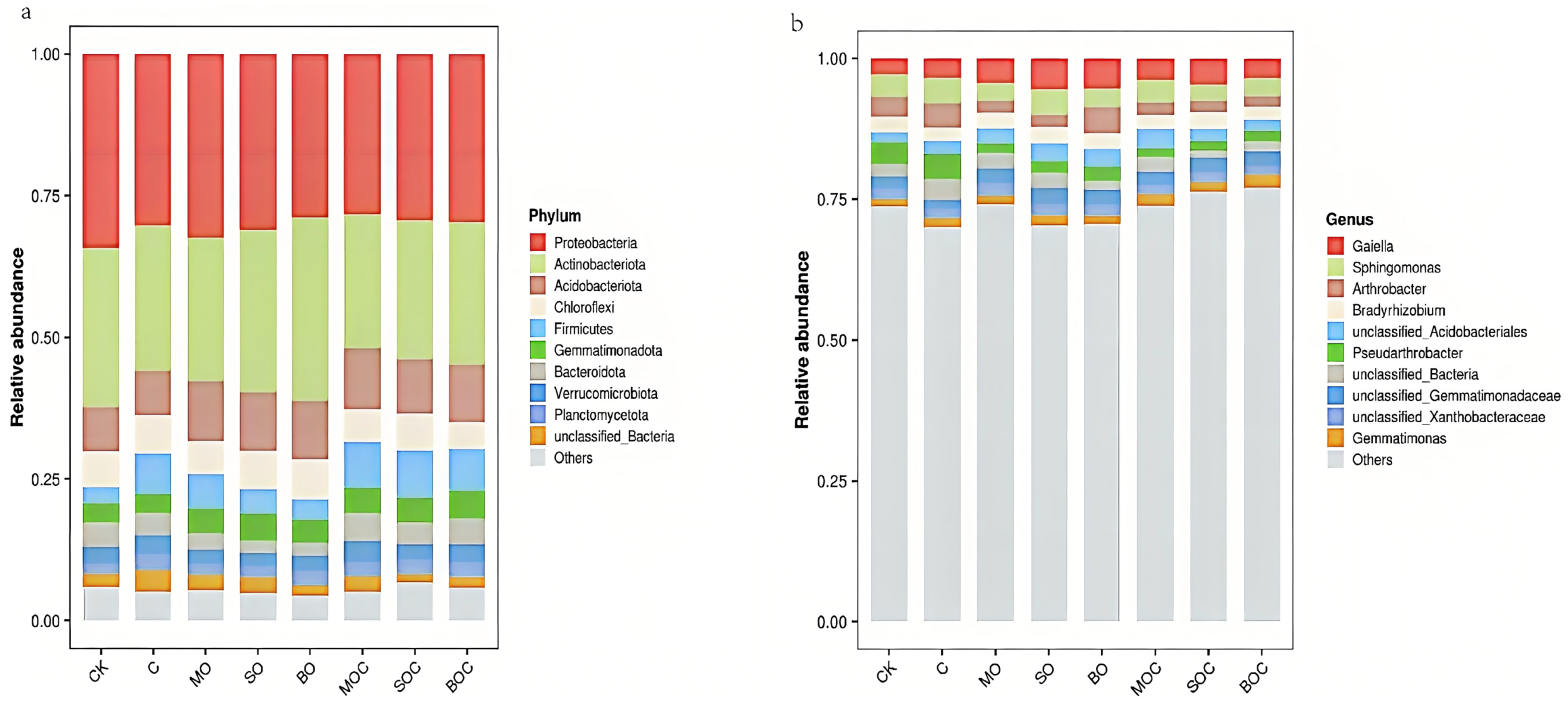
Stacked bar chart of relative abundance of bacterial groups at the phylum and genus levels at different fertilization regimes. The relative abundance of the top 10 species at the taxonomic level of the phylum **(a)** and genus **(b)** is displayed; the horizontal and vertical coordinates represent the sample name and relative abundance.

Community composition analysis further revealed that, compared with the sole application of organic fertilizer, the combined application of organic fertilizer and biochar increased the relative abundances of Actinobacteriota, Firmicutes, Gemmatimonadota, and Planctomycetota, while decreasing those of Proteobacteria and Verrucomicrobiota (Figure 8a). At the genus level (Figure 8b), the relative abundances of *Gaiella* and *Gemmatimonas* were increased, whereas those of *Arthrobacter* and *Pseudarthrobacter* were reduced.

### 3.6 Functional clustering of rhizosphere soil bacteria under different treatments

**Figure 9:**
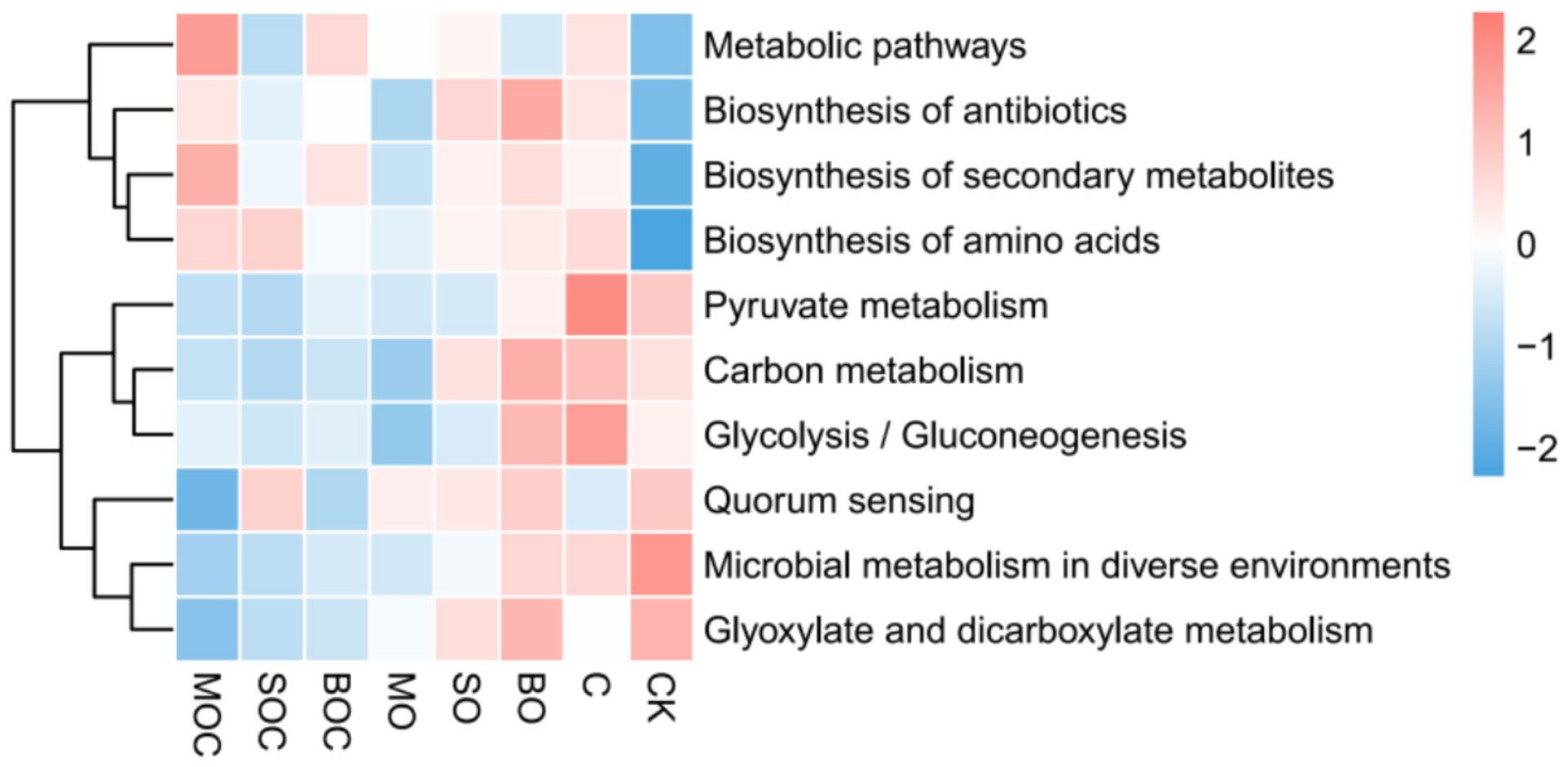
Heatmap of the top 10 predicted functional differences in rhizosphere soil under different combined applications of organic fertilizer and biochar. The *x*-axis represents different fertilization treatments (MOC, SOC, BOC, MO, SO, BO, CK), and the *y*-axis shows the major predicted metabolic pathways.

Subsequent functional clustering analysis of the rhizosphere soil after treatment (Figure 9) showed that the combined application of organic fertilizer and biochar resulted in a distinct clustering pattern of the ten most significantly altered predicted microbial functions. Compared with the individual application treatments (MO, SO, BO) and the control (CK), the combined treatment displayed a coordinated shift in several functional categories, mainly involving microbial metabolic pathways, secondary metabolite biosynthesis, and quorum sensing related functions. Overall, these results suggest that the combined application of organic fertilizer and biochar is associated with noticeable changes in the predicted functional composition of the rhizosphere microbiome, particularly in metabolism and communication related pathways.

### 3.7 Correlation analysis between rhizosphere soil bacteria and rapeseed traits

**Figure 10:**
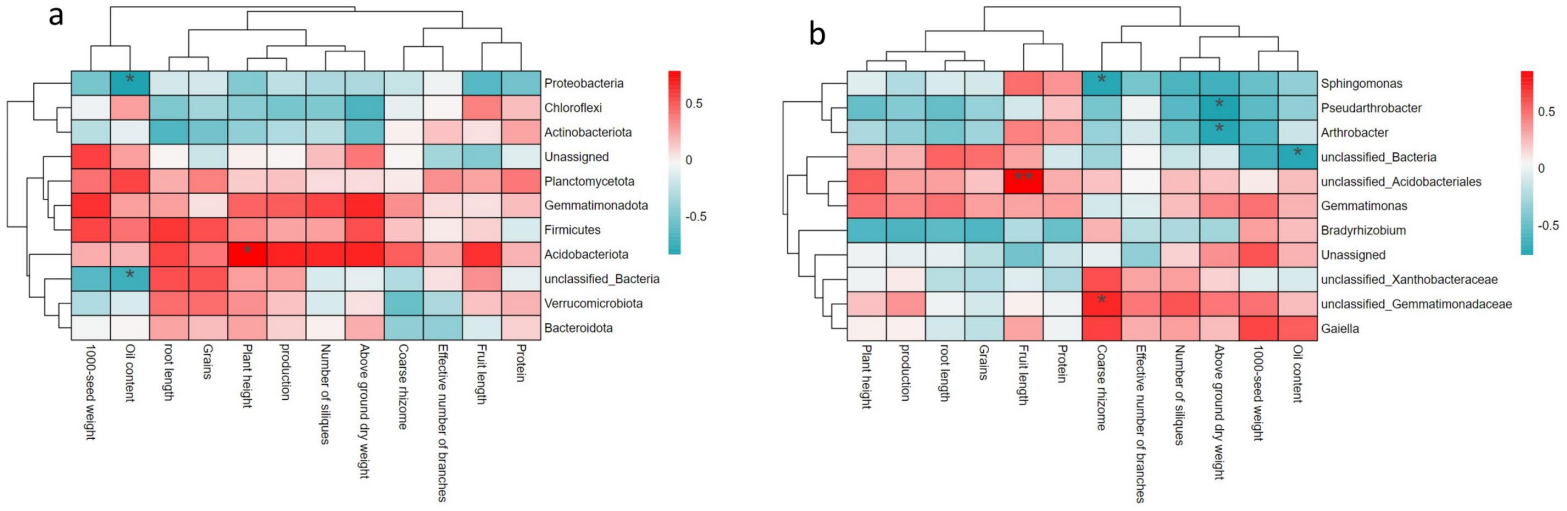
Correlation between traits, yield, and quality of rapeseed and dominant bacteria at the phylum and genus levels. **(a)** Shows the phylum level, while **(b)** shows the genus level. ^*^ indicates *p* < 0.05, ^**^ indicates *p* < 0.01, and ^***^ indicates *p* < 0.001.

Correlation analysis between the dominant bacterial phyla and genera in the rhizosphere soil of rapeseed and its agronomic traits, yield, and quality indicators revealed the following:

At the phylum level (Figure 10a.), Proteobacteria and unclassified bacteria were significantly negatively correlated with oil content, while Acidobacteriota showed a significant positive correlation with plant height. Rapeseed yield, oil content, and protein content were positively correlated with several bacterial phyla. Specifically, yield was positively associated with Planctomycetota, Gemmatimonadota, and Firmicutes; oil content was positively correlated with Chloroflexi, Acidobacteriota, and others; and protein content showed positive correlations with Actinobacteria, Acidobacteriota, and multiple other groups.

At the genus level (Figure 10b), Correlation analysis revealed that unclassified bacteria was significantly negatively correlated with oil content, whereas unclassified Acidobacteriales showed a strong positive association with plant height. Unclassified Gemmatimonadaceae was significantly positively correlated with stem diameter. Yield exhibited positive correlations with genera including *Gemmatimonas* and *Gaiella*, while oil content was positively associated with *Bradyrhizobium* and *Gaiella*. In addition, protein content showed positive correlations with unclassified Acidobacteriales, *Gemmatimonas*, and several other taxa. Conversely, *Sphingomonas*, *Pseudarthrobacter*, and *Arthrobacter* were significantly negatively correlated with aboveground dry biomass or stem diameter.

Overall, these correlation results indicate that variations in the relative abundance of certain bacterial phyla and genera in the rhizosphere are statistically associated with rapeseed agronomic traits, yield, and quality in dicators.

### 3.8 Correlation analysis between rhizosphere soil bacteria and soil chemical properties

Correlation analysis between dominant bacterial taxa at the phylum and genus levels in the rhizosphere of rapeseed and soil chemical properties revealed a close relationship between microbial community structure and soil nutrient status.

**Figure 11:**
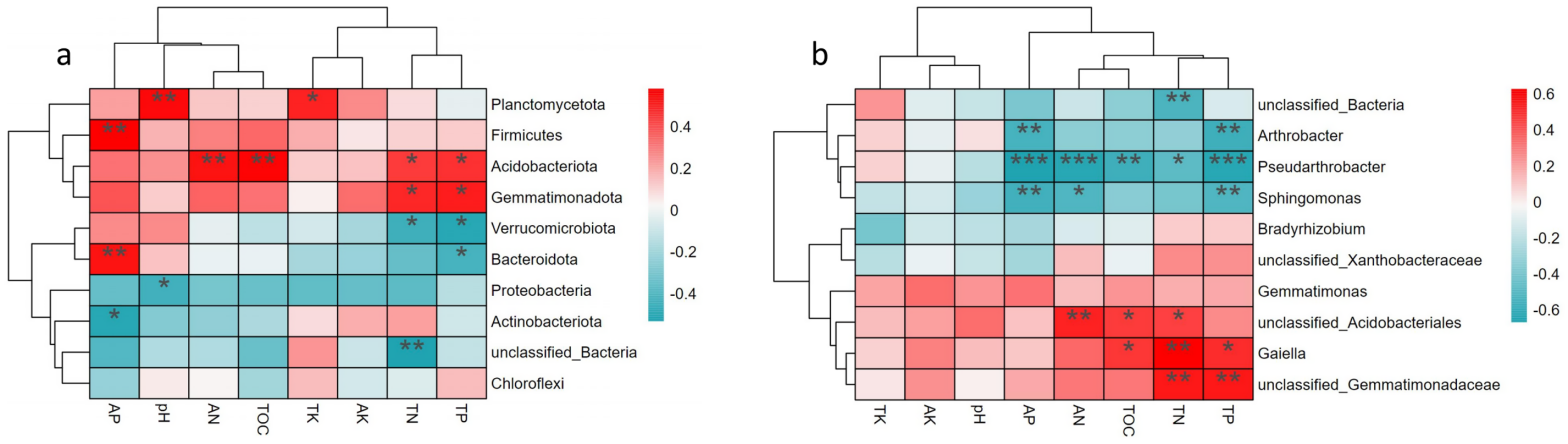
Correlation between soil chemical properties and dominant bacteria at the phylum and genus levels. **(a)** Phylum level. **(b)** Genus level.

At the phylum level (Figure 11a), Planctomycetota was significantly positively correlated with total potassium and soil pH, respectively. Firmicutes showed a highly significant positive correlation with available phosphorus. Acidobacteriota was significantly positively correlated with total nitrogen and total phosphorus, and highly significantly correlated with available nitrogen and organic matter. Gemmatimonadota also showed significant positive correlations with total nitrogen and total phosphorus. Bacteroidota was highly significantly positively correlated with available phosphorus but significantly negatively correlated with total phosphorus. In contrast, Proteobacteria showed a significant negative correlation with pH, while unclassified bacteria was highly significantly negatively correlated with total nitrogen.

At the genus level (Figure 11b), unclassified Acidobacteriales, *Gaiella*, and unclassified Gemmatimonadaceae all showed significant or highly significant positive correlations with nutrient indicators such as total nitrogen and total phosphorus. In contrast, *Pseudarthrobacter*, *Sphingomonas*, and *Arthrobacter* were significantly or highly significantly negatively correlated with multiple soil nutrient indicators.

Taken together, these correlation patterns demonstrate that variations in the relative abundance of specific rhizosphere bacterial phyla and genera are significantly associated with soil chemical properties, particularly nitrogen- and phosphorus-related indicators, highlighting a close statistical linkage between rhizosphere microbial community composition and soil nutrient status under different treatments.

## 4 Discussion

### 4.1 Context-dependent responses of rapeseed growth and quality to combined organic fertilizer and biochar application

The responses of rapeseed growth, yield, and quality to the combined application of organic fertilizers and biochar were strongly dependent on the source of organic fertilizer. Such context dependency is consistent with previous studies showing that organic amendments differ substantially in nutrient composition, decomposition rate, and C/N ratio, which collectively influence nutrient supply patterns and plant resource allocation during the growing season (Hu et al., 2024).

In rapeseed, excessive or imbalanced nitrogen inputs have been reported to favor vegetative growth at the expense of seed quality, particularly oil and protein accumulation (Hamzei, 2011).

Regarding the application of sheep manure organic fertilizer combined with biochar, the observed increase in rapeseed oil content accompanied by a decline in protein content may be partly attributed to biochar-mediated changes in nitrogen availability. Biochar residues are characterized by a porous structure, large specific surface area, abundant surface functional groups, and stable aromatic carbon fractions, which can adsorb and retain inorganic nitrogen, including NH₄^+^ and NO₃^−^, and alter nitrogen mineralization–immobilization dynamics in soil (Clough et al., 2013; Nguyen et al., 2017; Phillips et al., 2022). These processes may temporarily reduce plant-available nitrogen during seed filling, thereby limiting nitrogen allocation to seed protein synthesis. In addition, because biochar partially replaced sheep manure organic fertilizer in the combined treatment, the amount of manure-derived nitrogen input may have been reduced compared with the sole sheep manure treatment. The negative relationship between oil and protein content under sheep manure-related treatments may further reflect the physiological trade-off between carbon allocation to lipid biosynthesis and nitrogen allocation to protein synthesis during seed development. Previous studies on rapeseed/canola have shown that increasing nitrogen supply generally increases seed protein concentration but often decreases seed oil concentration, indicating an inverse relationship between these two quality traits (Brennan et al., 2000; Gu et al., 2023; Peltonen-Sainio et al., 2011). Therefore, the increase in oil content and decrease in protein content under the SOC treatment may not be caused by biochar residue alone, but rather by the combined effects of biochar-regulated nitrogen retention, reduced manure-derived nitrogen input, and the intrinsic carbon–nitrogen allocation trade-off during seed development. In contrast, the co-application of macadamia nutshell or sugarcane bagasse organic fertilizers with biochar resulted in simultaneous improvements in TSW, oil content, and protein content compared to the sole application of organic fertilizer. These source-dependent responses are consistent with previous findings, suggesting that specific co-applications of organic fertilizer and biochar can optimize nutrient supply patterns to satisfy the requirements for both carbon and nitrogen metabolism in rapeseed (Khan et al., 2022).

The contrasting responses observed among different organic fertilizer–biochar combinations suggest that biochar does not exert a uniform “growth-promoting” effect but rather modulates nutrient availability and release efficiency in a manner contingent on amendment characteristics (Shah et al., 2023). Optimizations in certain quality traits without corresponding yield increases further indicate potential trade-offs between biomass production and seed composition, a phenomenon that has also been reported in previous studies under altered nutrient regimes (Libutti et al., 2023). When evaluating the agronomic effects of biochar co-application, it should be considered within the context of specific organic fertilizer sources and management objectives, rather than assuming universally positive outcomes.

### 4.2 Short-term regulation of soil chemical properties under combined amendments

Soil organic matter content and nutrient status are widely regarded as fundamental indicators of soil quality and fertility. Previous studies have shown that biochar can stabilize carbon derived from organic fertilizers through interactions with soil organic matter and mineral components, thereby influencing short term soil carbon dynamics (Butterly et al., 2020; Schulz and Glaser, 2012). However, such effects are strongly context dependent. In the present study, the combined application of sugarcane residue organic fertilizer and biochar did not result in a significant increase in soil organic matter compared with organic fertilizer applied alone, indicating that the effects of biochar and organic fertilizer combinations on soil organic matter are jointly regulated by organic fertilizer source, biochar properties, climatic conditions, and fertilization practices (Eskelinen and Harrison, 2015).

Building on this, changes in soil nutrient status provide further insight into the short-term effects of combined amendments. Consistent with previous findings, the application of organic fertilizers, either alone or in combination with biochar, increased total soil nitrogen, phosphorus, and potassium contents (Kimani et al., 2021; Phares and Akaba, 2022). Long term field studies have further demonstrated that, compared with organic fertilizer alone, the co-application of biochar and organic fertilizer generally exerts a more pronounced effect on soil nutrient accumulation, particularly for nitrogen and phosphorus. This response may be attributed to the buffering capacity of background soil nutrients, the limited direct nutrient contribution of biochar, and its ability to adsorb nutrients and regulate their release (Cheng et al., 2025).

Beyond changes in total nutrient pools, biochar co-application may also influence nutrient availability in soil solution by improving soil structure and enhancing nutrient retention capacity (Bednik et al., 2023). However, soil phosphorus responses to biochar application are complex. Studies have shown that long-term, high-rate biochar application has little effect on total soil phosphorus but may substantially reduce available phosphorus due to strong sorption processes (Schmidt et al., 2017). In addition, nitrogen and potassium in biochar are predominantly present in organic or poorly soluble forms, and their transformation into plant available forms is largely governed by soil environmental conditions and microbial activity. This may partly explain the limited changes in available nitrogen and potassium observed under certain treatments in the present study (Li et al., 2021).

In the present study, the combined application of organic fertilizer and biochar was more effective in increasing soil available phosphorus than either amendment applied alone. This response may reflect the complementary roles of organic fertilizers as direct phosphate (PO_4_^3 -^)sources and biochar as a nutrient-retaining matrix. The large specific surface area and porous structure of biochar facilitate the adsorption and retention of phosphate released during organic fertilizer decomposition, thereby reducing nutrient losses and improving phosphorus use efficiency (Meng et al., 2023). In addition, biochar-induced shifts in microbial community structure may further promote phosphorus transformation, as microbial decomposition processes can facilitate the conversion of organic or inert phosphorus pools into more bioavailable forms (Gong et al., 2020).

In contrast, biochar applied alone significantly reduced soil available phosphorus. This finding is consistent with previous reports indicating that the activated carbon fraction of biochar exhibits strong physical adsorption capacity for phosphate, and its accumulation in soil can enhance surface sorption of PO_4_^3 -^, thereby constraining phosphorus availability (Wang et al., 2025). Taken together, at the short-term scale, integrating biochar with organic fertilizer may help to avoid unintended limitations on soil phosphorus availability.

### 4.3 Effects of combined application of organic fertilizer and biochar on bacterial communities in the rhizosphere soil of rapeseed

Rhizosphere bacterial communities play a crucial role in soil nutrient cycling and crop growth performance (Meng et al., 2013). Previous studies have demonstrated that the combined application of biochar and organic fertilizer can improve the microbial habitat and increase the diversity and abundance of bacterial communities (Hou et al., 2024; Yuan et al., 2021). Consistent with these findings, the present study showed that combined application treatments were significantly associated with higher bacterial diversity and pronounced shifts in community composition in the rhizosphere soil of rapeseed. Recent studies on biochar-based amendments further support this interpretation by showing that biochar can regulate soil–microbe–plant interactions under different stress conditions. For instance, bacterial-charged biochar has been reported to promote plant growth and alleviate microplastic toxicity by modifying soil metabolism and microbial communities, whereas nano-engineered biochar composites enhanced soil microbial interactions and maize transcriptomic pathways related to cadmium detoxification (Afzal et al., 2025; Yasin et al., 2025). Although these studies were conducted under different stress scenarios, they support the broader view that biochar-based amendments can coordinate nutrient regulation, microbial community restructuring, and plant performance. Therefore, the shifts in bacterial diversity and community composition observed in the present study may represent an important biological pathway through which organic fertilizer–biochar co-application regulates rapeseed performance in acidic red soil.

These effects may be attributed to the complementary properties of biochar and organic fertilizer. Biochar provides porous microenvironments that facilitate microbial colonization, while organic fertilizers supply readily available carbon sources and essential nutrients (N, P, and K) for microbial growth, in addition, their combined application may buffer soil pH and prolong nutrient availability (Duminda et al., 2016; Fernández-Calviño et al., 2018; Lehmann et al., 2011; Ortiz and Sansinenea, 2022; Pan et al., 2020). This is consistent with the general view that the porous structure of biochar can provide microbial habitats and alter soil physical and chemical conditions, thereby shaping microbial diversity and activity. Biochar has been reported to support microbial habitats through its porous structure and by promoting stable soil micro-aggregates (Bolan et al., 2024).

Several bacterial phyla previously reported to be involved in soil organic matter turnover, nutrient cycling, and plant microbe interactions responded to the combined application treatments, including Firmicutes, Actinobacteriota, Bacteroidota, Verrucomicrobiota, Planctomycetota, Gemmatimonadota, Proteobacteria, and Chloroflexi (Asaf et al., 2020; Bergmann et al., 2011; Ferguson et al., 2020; Hsouna et al., 2024; Long et al., 2017; López-Mondéjar et al., 2020; Navarrete et al., 2012; Peng et al., 2024; Trivedi et al., 2016; Trivedi et al., 2020), while *Bacillus*, belonging to Firmicutes, can form biofilms on crop roots and play an important role in controlling crop diseases (Zhu et al., 2020). Under the MOC treatment, the enrichment of Acidobacteriota, Bacteroidota, and Verrucomicrobiota may be associated with the combined effects of macadamia nutshell-derived organic substrates and biochar-derived microhabitats. Macadamia nutshells are lignocellulosic residues rich in cellulose, hemicellulose, and lignin, which may provide diverse carbon substrates during decomposition (Khan et al., 2023). Acidobacteriota are metabolically diverse and have been associated with organic carbon utilization and lignocellulosic plant biomass degradation (Kalam et al., 2020; Rawat et al., 2012). Bacteroidota are well known for their capacity to degrade complex polysaccharides and participate in plant residue decomposition (Huang et al., 2023; Thomas et al., 2011).

Verrucomicrobiota have been frequently detected in soil and rhizosphere environments and have been linked to the degradation of plant-derived organic matter (Bünger et al., 2020; Rakitin et al., 2024). Therefore, the enrichment of these phyla under MOC may indicate enhanced rhizosphere carbon turnover and nutrient transformation driven by the interaction between macadamia nutshell organic fertilizer and biochar-modified microhabitats.

Under SOC, the enrichment of Firmicutes and Patescibacteria may be related to the manure-derived organic carbon and nutrient inputs from sheep manure organic fertilizer, as manure amendments can alter soil physicochemical properties and reshape microbial community structure (Yu et al., 2024). Firmicutes include many spore-forming and stress-tolerant bacteria, such as *Bacillus*, which can survive under fluctuating environmental conditions and contribute to organic matter decomposition, nutrient mobilization, and plant growth promotion (Etesami et al., 2023). Patescibacteria generally possess small genomes and limited biosynthetic capacities, and many members are considered to rely on symbiotic or host-associated lifestyles (Chaudhari et al., 2021). Therefore, the SOC treatment may have promoted bacterial groups adapted to manure-derived nutrient pulses and biochar-associated microenvironments.

Under BOC, the enrichment of Gemmatimonadota, Myxococcota, and Bdellovibrionota may be explained by changes in nutrient retention, phosphorus cycling, and microbial food-web interactions. Biochar can create porous microhabitats and improve nutrient retention, thereby providing favorable microsites for specific microbial groups (Bolan et al., 2024). Members of Gemmatimonadota, especially *Gemmatimonas*, have been reported as polyphosphate-accumulating microorganisms, suggesting their potential involvement in phosphorus transformation and storage (Zhang et al., 2003). In addition, Myxococcota and Bdellovibrionota include predatory bacteria that can regulate bacterial prey populations and influence microbial community structure (Williams and Chen, 2020). Therefore, their enrichment under BOC may indicate that sugarcane bagasse organic fertilizer combined with biochar promoted not only nutrient cycling but also top-down regulation within the rhizosphere microbial food web.

The enrichment of nitrogen oxidizers in CK may be explained by the lower organic carbon input and weaker stimulation of heterotrophic bacteria in the control soil. In the absence of organic amendments, bacteria associated with inorganic nitrogen oxidation may occupy a relatively higher relative abundance. Therefore, the enrichment of nitrogen oxidizers in CK should be interpreted as a relative increase caused by reduced competition from amendment-stimulated heterotrophs, rather than direct evidence of higher absolute abundance or nitrification activity (Fierer et al., 2007).

The increase in Actinobacteriota under combined application treatments may be attributed to improved substrate supply and microbial habitat conditions. Organic fertilizers provide decomposable carbon and nutrients, while biochar improves soil aeration, pH buffering, nutrient retention, and microbial microsites. Since Actinobacteria are widely involved in complex organic matter decomposition, nutrient cycling, and plant growth promotion, their enrichment may have contributed to improved soil fertility and rhizosphere functioning under combined amendment treatments (Barka et al., 2016).

The decrease in the relative abundance of Proteobacteria and Verrucomicrobiota under some combined treatments should be interpreted cautiously. Because sequencing data represent relative abundance, a decline in these taxa does not necessarily indicate a decrease in their absolute population size. Instead, it may reflect the stronger enrichment of other bacterial groups, such as Actinobacteriota, Firmicutes, and Gemmatimonadota, under combined amendment conditions (Gloor et al., 2017).

At the genus level, the increased relative abundances of *Gaiella* and *Gemmatimonas* under combined treatments may be associated with improved nutrient availability, organic matter turnover, and phosphorus transformation. In particular, *Gemmatimonas* has been reported as a polyphosphate-accumulating microorganism, suggesting its potential role in phosphorus storage and nutrient cycling. In contrast, the decreases in *Arthrobacter* and *Pseudarthrobacter* may reflect competitive replacement by bacterial taxa better adapted to nutrient-enriched and biochar-modified rhizosphere conditions (Zhang et al., 2003).

The negative correlation between Proteobacteria and oil content should be regarded as a statistical association rather than direct evidence that Proteobacteria inhibited oil accumulation. Proteobacteria include many taxa involved in nitrogen transformation and rapid responses to nutrient availability. Therefore, their negative relationship with oil content may reflect soil conditions with stronger nitrogen turnover, as higher nitrogen availability in rapeseed/canola commonly increases seed protein concentration but decreases oil concentration (Brennan et al., 2000).

Overall, changes in bacterial genera may partly explain the improvement of rapeseed agronomic traits through their roles in nutrient cycling, organic matter decomposition, phosphorus transformation, stress resistance, and plant growth promotion. For example, *Gaiella* and *Gemmatimonas* may contribute to nutrient cycling, *Bradyrhizobium* may be associated with nitrogen-related processes, and *Bacillus* is widely recognized for promoting plant growth, nutrient mobilization, biofilm formation, and disease suppression. These results suggest that combined application improved rapeseed performance through coordinated restructuring of rhizosphere bacterial communities rather than through a single bacterial group (Trivedi et al., 2020).

## 5 Conclusion

This study demonstrates that the co-application of organic fertilizers and biochar significantly influences rapeseed agronomic traits, soil chemical properties, and rhizosphere microbial communities in acidic red soils. However, rather than exhibiting a uniform growth-promoting effect, the outcomes of these integrated amendments are strongly source-dependent, reflecting distinct interactions between the specific organic fertilizer and biochar.

Comparative analysis reveals that the co-application of biochar does not consistently outperform individual organic amendments in terms of crop yield. While sugarcane bagasse-based organic fertilizer combined with biochar (BOC) significantly increased TSW and overall yield compared to its sole application (BO), other combinations yielded contrasting agronomic responses. Specifically, single applications of macadamia nutshell (MO) and sheep manure (SO) organic fertilizers resulted in higher per-plant yields than their biochar-supplemented counterparts (MOC and SOC). Instead of driving yield, SOC and MOC treatments shifted plant resource allocation toward quality traits, favoring the accumulation of seed oil and protein. This indicates a clear trade-off between biomass production and seed quality improvement, dictated by the origin of the organic amendment.

Overall, rational amelioration strategies for acidic red soils must be tailored to specific agronomic management objectives rather than assuming a universal superiority of co-application. BOC serves as a practical short-term strategy for maximizing rapeseed yield, whereas SOC and MOC may be prioritized when the primary goal is enhancing seed nutritional quality. Future multi-season field studies are required to validate the long-term stability of these source-specific responses and to refine precision fertilization frameworks.

## Data Availability

The datasets generated or analyzed during this study are available from the corresponding author upon reasonable request. The sequencing data have been deposited in the NCBI Sequence Read Archive (SRA) under the BioProject accession number PRJNA1345503.
